# Prognostic Fifteen-Gene Signature for Early Stage Pancreatic Ductal Adenocarcinoma

**DOI:** 10.1371/journal.pone.0133562

**Published:** 2015-08-06

**Authors:** Dung-Tsa Chen, Ashley H. Davis-Yadley, Po-Yu Huang, Kazim Husain, Barbara A. Centeno, Jennifer Permuth-Wey, Jose M. Pimiento, Mokenge Malafa

**Affiliations:** 1 Department of Biostatistics and Bioinformatics, Moffitt Cancer Center & Research Institute, Tampa, FL, 33612, United States of America; 2 Department of Gastrointestinal Oncology, Moffitt Cancer Center & Research Institute, Tampa, FL, 33612, United States of America; 3 Department of Applied Mathematics and Institute of Statistics, National Chung Hsing University, Taichung City, Taiwan; 4 Department of Anatomic Pathology, Moffitt Cancer Center & Research Institute, Tampa, FL, 33612, United States of America; 5 Department of Cancer Epidemiology, Moffitt Cancer Center & Research Institute, Tampa, FL, 33612, United States of America; Centro Nacional de Investigaciones Oncológicas (CNIO), SPAIN

## Abstract

The outcomes of patients treated with surgery for early stage pancreatic ductal adenocarcinoma (PDAC) are variable with median survival ranging from 6 months to more than 5 years. This challenge underscores an unmet need for developing personalized medicine strategies to refine the current treatment decision-making process. To derive a prognostic gene signature for patients with early stage PDAC, a PDAC cohort from Moffitt Cancer Center (n = 63) was used with overall survival (OS) as the primary endpoint. This was further evaluated using an independent microarray cohort dataset (Stratford et al: n = 102). Technical validation was performed by NanoString platform. A prognostic 15-gene signature was developed and showed a statistically significant association with OS in the Moffitt cohort (hazard ratio [HR] = 3.26; p<0.001) and Stratford et al cohort (HR = 2.07; p = 0.02), and was independent of other prognostic variables. Moreover, integration of the signature with the TNM staging system improved risk prediction (p<0.01 in both cohorts). In addition, NanoString validation showed that the signature was robust with a high degree of reproducibility and the association with OS remained significant in the two cohorts. The gene signature could be a potential prognostic tool to allow risk-adapted stratification of PDAC patients into personalized treatment protocols; possibly improving the currently poor clinical outcomes of these patients.

## Introduction

Pancreatic cancer is the fourth leading cause of cancer death in the United States with an estimated 38,000 deaths in 2013[1]. Only twenty percent of patients with pancreatic ductal adenocarcinoma (PDAC) have stage I and II disease and are candidates for potential curative treatment that typically includes surgical resection and adjuvant chemotherapy with or without radiation treatment. While the five-year survival rate for curative-intent surgical resection for pancreatic cancer is 15 to 30% [2, 3], there is substantial individual variation. Currently, the only accepted prognostic factor guiding treatment decisions for both surgeons and oncologists is the AJCC TNM staging. However, the prognostic performance of AJCC TNM staging for more than 80% of patients with resected pancreatic cancer (Stages IB, IIA, and IIB) is very poor, with the survival curves being virtually identical[4]; therefore, the current practice is to uniformly treat all patients with stage I and II PDAC with surgical resection followed by adjuvant therapy. This approach results in potential undertreatment with surgical resection for patients who are at high risk of early recurrence and overtreatment with adjuvant therapy for patients who are at low risk of recurrence. The core obstacle to personalized management strategies is the lack of definitive prognostic biomarker(s) to identify stage I and II PDACs with a high probability of occult metastases and corresponding poorer clinical outcomes. Better prognostic tools are needed to identify patients predicted to be at high or low risk of recurrence to help guide treatment decisions for medical and radiation oncologists as well as pancreatic surgeons.

Recent advances in genomic cancer research have led to numerous biomarker discoveries; some have been validated and have become clinical assays to help improve patient care. For example, Oncotype DX, a 21-gene signature has been used to predict breast cancer recurrence in patients with node negative disease, such that patients with a high risk score are recommended for receiving adjuvant chemotherapy[5, 6]. In the management of advanced non-small cell lung cancer, epidermal growth factor receptor (EGFR) mutation testing is routinely used to guide treatment plans[7]. While there are successful stories of biomarker application for clinical use in breast cancer[5], lung cancer[8], colorectal cancer[9], and melanoma[10], sadly, the clinical application of biomarkers in PDAC is very limited. For example, serum CA 19–9[11], a FDA-approved biomarker (since 1980s), has been used for prognosis in PDAC even though at least 10% of patient do not express it and metabolic abnormalities such as hyperbilirubinemia can affect it significantly. As such, there is an unmet need to develop reliable and robust biomarkers such as gene signatures to better predict outcomes and to help tailor treatment plans (such as use of surgery versus systemic therapy).

In response to this unmet need, we have analyzed microarray data from a Moffitt cohort of 63 patients with early stage PDAC (stage IB, IIA and IIB) and developed a prognostic 15-gene signature to predict overall survival (OS). We hypothesize that high risk for poor clinical outcomes in early-stage PDAC are reflected by specific transcriptomic features from this 15-gene signature.

## Materials and Methods

### Study cohorts

The study aimed to utilize the Moffitt cohort to develop a prognostic gene signature for PDAC patients. Since the sample size was not very large (n = 63), an external cohort (Stratford et al; n = 102) was used for validation. We targeted a large effect size (HR>3) in order to reach 80% power or higher for a dichotomized gene signature score. Specifically, for the Moffitt cohort, with 42 events and assumption of 1:1 ratio of low and high risk groups by the gene signature, the power could reach at least 80% to detect a HR of 2.6 with type I error controlled at 5%. For the Stratford et al cohort (66 events), a ratio of 1:1 for low and high risk groups by the gene signature could reach 98% to detect a HR of 3. For a ratio of 1:3, the power was 80%. Power calculation was based on ‘powerSurvEpi’ R package. Analysis of combination of the signature with TNM staging system was also performed, but mainly for exploration due to potential small sample size issue in subgroup. However, if the analysis showed consistent patterns in both cohorts, the results would be reported and discussed to warrant further study. A flow chart of study cohorts and data analysis was provided in S1 Fig.

### Moffitt cohort

This is a retrospective microarray study of resected PDAC samples at Moffitt Cancer Center constituting the primary dataset to develop the gene signature. The sample cohort, consisting of fresh frozen macrodissected tumor tissues from 63 patients with early stage PDAC, was collected and profiled from 2006 to 2011 under the Moffitt Total Cancer Care protocol and the research protocol MCC 17779 approved by Moffitt’s Scientific Review Committee and the Institutional Review Board (IRB #10.07.0008). The inclusion criteria were patients who underwent surgery for early stage/resectable (stage I and II) pancreatic cancer, that were consented for the TCC protocol and that had complete gene expression analysis. Exclusion criteria were patients found to be metastatic at presentation or patients without gene expression data. Patient information was anonymized and de-identified. Gene expression data were generated using Rosetta/Merck Human RSTA Custom Affymetrix 2.0 microarray (http://www.ncbi.nlm.nih.gov/geo/query/acc.cgi?acc=GSE57495) The median follow-up time was 21 months (21 samples were from patients who were alive and 42 samples were from patients who had died). In other words, two thirds (67% = 42/63) of patient died in the follow-up period. Of these 42 patients, 9 (21% = 9/42) died from other diagnosis not cancer recurrence (n = 8) or unknown disease status (n = 1). However, due to nature of the retrospective study and limitation of registration of recurrent disease, patient died caused by recurrent disease may not be diagnosed. Distribution of survival time was not significantly different between patients who died from other causes and patients who died by cancer recurrence (p = 0. 71; mean of survival time: 16.2 (standard deviation (SD) = 12.37) versus 18.2 (SD = 8.62) months for other causes and cancer recurrence, respectively). Clinical predictors included TNM stage, T stage, N stage, gender, histology grade, and age at diagnosis (Table 1).

**Table 1:** Descriptive statistics of clinical predictors in the Moffitt PDAC cohort (n = 63).

TNM stage	IB	13
	IIA	17
	IIB	33
T stage	T1	2
	T2	14
	T3	47
N stage	N0	30
	N1	32
	NA	1
Gender	Female	30
	Male	33
Histology grade	Well-Differentiated	6
	Moderate-Differentiated	35
	Poor-Differentiated	18
	NA	4
Age at diagnosis	youngest	24 years
	1st quartile	60.5 years
	median	68 years
	3rd quartile	74 years
	oldest	86 years

One independent microarray dataset in pancreatic cancer was included to validate the gene signature: *Stratford et al[12] localized PDAC study* (http://www.ncbi.nlm.nih.gov/projects/geo/query/acc.cgi?acc=GSE21501). The Stratford et al cohort[12] examined 132 patients with PDAC for genomic profiling. Among these patients, there were 102 patients with OS available. These patients (n = 102) were used to validate our gene signature. Microarray data were generated using Agilent-014850 Whole Human Genome Microarray 4x44K G4112F Array. We evaluated if the signature could predict OS in this cohort.

### NanoString validation

RNA samples of fresh frozen macrodissected tumor tissues from 53 PDAC patients in the Moffitt cohort (a subset of the 63 PDAC patients) were used to measure gene expression of the 15-gene signature in the NanoString platform. The NanoString Assays were performed with 150-ng aliquots of RNA using the NanoString nCounter Analysis system. Nineteen invariant genes were selected to serve as house-keeping genes for normalization (S1 Table). After codeset hybridization overnight, the samples were washed and immobilized to a cartridge using the NanoString nCounter Prep Station. Cartridges were scanned in the nCounter Digital Analyzer at 555 fields of view for the maximum level of sensitivity. Gene expression was normalized using NanoStringNorm R package. Specifically, background correction was performed using the negative control at the cutoff of mean + 2 standard deviation. House-keeping genes were used to for normalization based on geometric mean.

### Statistical Analysis

#### Data normalization

Different normalization methods could affect the results. However, there is no gold standard of which normalization to be best used. The problem gets more complicated when both cohorts used different microarray platforms. Each platform favors certain types of normalization to account for its unique microarray design. For Affymetrix genechip, RMA [13] is a common approach for normalization while the Loess method[14] is often used for the Agilent microarray. For this reason, both normalizations were used for the study.

#### Development of a prognostic gene signature

The goal was to develop a prognostic gene signature. We used OS as the primary endpoint for analysis in the Moffitt cohort as the training cohort. Instead of performing supervised whole genome analysis, the first step was to use sparse PCA [15, 16] to filter out most genes with small variation. The remaining genes were analyzed using univariate analysis of Cox proportional hazards model to identify genes associated with OS at the 25% level of the false discovery rate [17]. Genes associated with OS were stratified into two subsets: (1) genes with higher expression associated with poor survival and (2) genes with lower expression associated with poor survival. The PC1 scoring system below was used for each gene subset for further analysis.

#### PC1 scoring system

An overall risk score was generated by principal component analysis (PCA) to reflect the combined effect of a gene signature. Specifically, we used the first principal component (PC1) to represent the overall expression level for the signature. That is, PC1, defined as ∑*w*_*i*_ *x*_*i*_, is a weighted average expression among all genes in the signature, where *x*_*i*_ represents gene *i* expression level, *w*_*i*_ is the corresponding weight (PC1’s loading coefficient for gene *i*) with $\sum w_{i}^{2}=1$, and the *w*_*i*_ values maximize the variance of ∑*w*_*i*_ *x*_*i*_. Prior to PCA, data were standardized by centering the mean and scaled by the standard deviation for each gene in the Moffitt cohort (training cohort). The standardized expression data were then used in PCA to generate principal components (PCs). Total variation of PC1 was examined to ensure at least two fold increase (compared to PC2) or more than 30% (both were based on our experiences in previous cancer studies [18, 19]). The PC1’s loading coefficients were then used (fixed) for both Moffitt and Stratford cohorts using their standardized expression data. The uniqueness of the PC1 scoring system is that it explains the largest total variation, likely linked to biological effect. More importantly, it integrates all molecular features into one score (efficient data reduction) for each patient, simplifying clinical decision-making. This approach has been used to derive various gene signatures previously [18–21]. For example, in our studies of lung and breast cancer[18, 19], we used PC1 to capture most gene signature information and this PC1 scoring approach was able to demonstrate the clinical association (e.g., cancer risk, prognosis, and prediction of chemotherapy) of gene signatures.

#### Association with OS and other clinical predictors

The influence of each gene signature was tested to see if the overall survival of two risk groups (high PC1 and low PC1) formed by a median-split of the PC1 score were statistically significantly different. The two-sided log-rank test was used to calculate p values. Evaluation of the median-split PC1 score, as an independent factor predicting PDAC prognosis, was done by clinical predictors including TNM stage (IB, IIA, and IIB), gender, histology grade, and age at diagnosis using multivariable Cox proportional hazards regression analysis.

#### External validation

One independent cohort was used to validate each gene signature. Due to the difference of microarray platforms in each cohort, gene level data were used for evaluation (a gene expression level was defined as an average of the expression level for a set of probe sets for the same gene; any probeset with a missing value was excluded). Before analysis, data were standardized by centering the mean and scaled by the standard deviation for each gene. The predetermined PC1’s loading coefficients (weights) derived from the Moffitt cohort were used to calculate the PC1 score for the independent cohort.

#### Evaluation of chance as a random gene signature

A resampling scheme was used to generate 100,000 random gene signatures to simulate chance significance for the two pancreatic cancer datasets. Specifically, this analysis was performed to evaluate whether a selected signature was not inferior to random signatures. Each random signature was generated by randomly selecting the same number of probesets as the selected signature from the Moffitt cohort; each random signature was then applied to the two datasets to determine the significance level. The p value, as a random gene signature for a targeted gene signature, was defined as the proportion of p values from the random signatures less than the observed p value from the targeted signature in both datasets. That is, it was defined as a joint probability of p values from the random signatures generated from the Moffitt cohort less than the observed p value in the Moffitt cohort and p values from the random signatures generated from the Stratford et al cohort less than the observed p value in the Stratford et al cohort. Instead of using all the probesets, only the probesets that passed sparse PCA filtering were used to evaluate the chance of the candidate signature as a random noise signature.

## Results

### A prognostic 15-gene signature

By analyzing early stage PDAC patients from the Moffitt cohort of 63 patients with PDAC, a prognostic 15-gene signature was developed to predict OS. Specifically, sparse principal component analysis[15, 16] screened out most genes (59,918 probesets) and yielded 689 probesets for 488 genes with non-zero coefficients using L1-penalty at the amount of 10^(-6)^. Further study of these genes using univariate analysis of Cox proportional hazards model showed 38 probesets (32 genes) associated with OS at 25% false discovery rate. The gene set was then separated into two subsets: (1) genes with higher expression associated with poor OS (18 probesets for 15 genes: C6orf15, CAPN8, HIST1H3H, IGF2BP3, KIF14, KRT6A, PMAIP1, PPBP, RTKN2, SCEL, SERPINB5, SLC2A1, SLC45A3, TMPRSS3, UCA1; S2 Table) and (2) genes with lower expression associated with poor OS (20 probesets for 17 genes). While both gene subsets showed the prognostic effect in the Moffitt cohort, the gene subset with lower expression associated with poor OS was unable to demonstrate the clinical association in later external validation (Stratford et al cohort[12]). Therefore, we focused on the gene subset with higher expression associated with poor OS, the 15-gene signature, for evaluation. Since the first principal component (PC1) explains most total variation (51%; S2 Fig), a scoring system was developed using the PC1 to derive a risk score to summarize gene expression for the signature.

### An independent prognostic predictor

**Fig 1:**
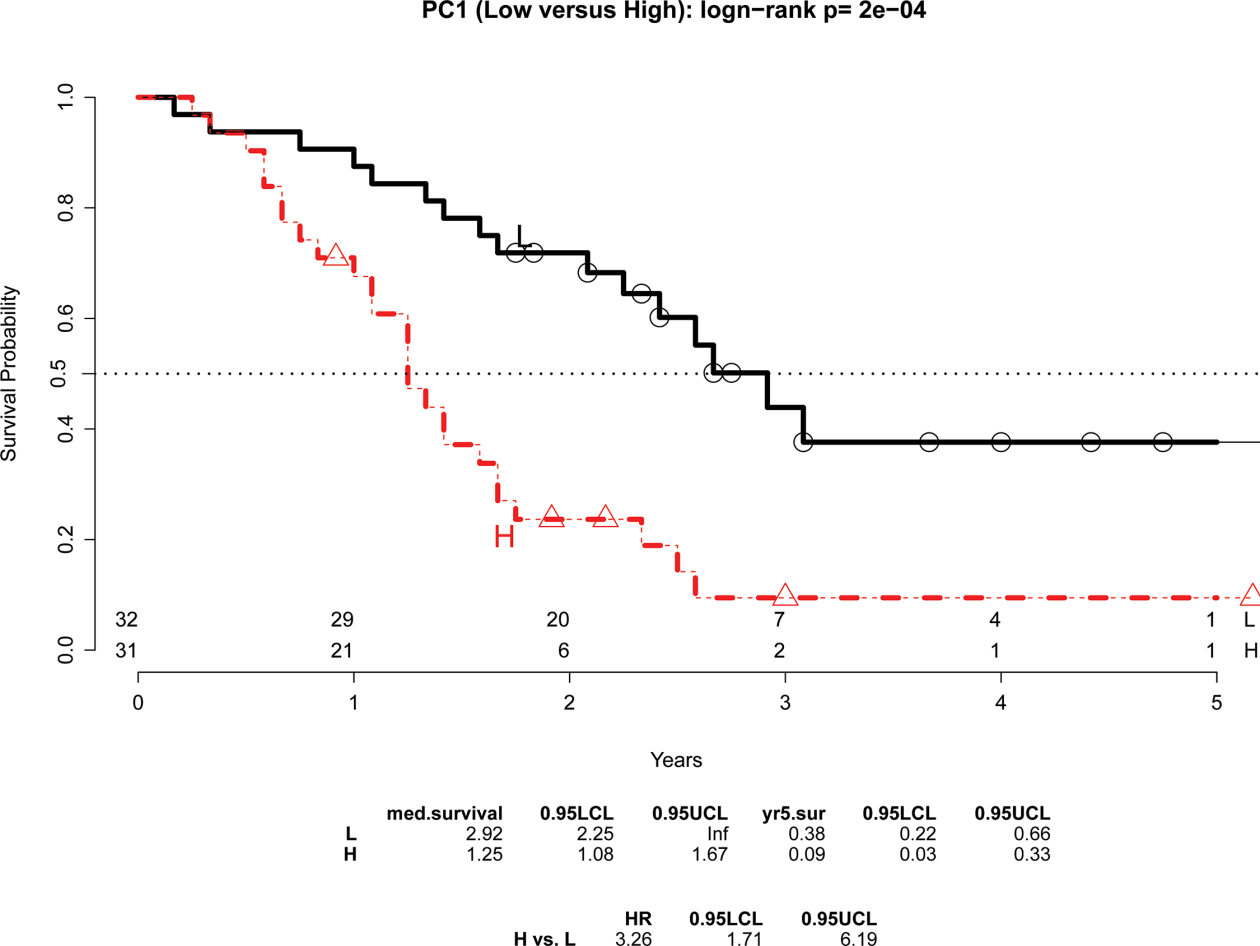
Association of the 15-gene signature with overall survival in the Moffitt cohort. A PC1 score was generated for each patient from the Moffitt cohort (n = 63) by principal component analysis to reflect the combined expression of the 15 genes. High and low PC1 groups were determined on the basis of a median split. Kaplan–Meier curves of overall survival are shown in the two groups. A statistically significant difference of the Kaplan–Meier survival curves between the high and low PC1 groups was determined by the two-sided log-rank test. The number of patients at risk is listed below the survival curves.

The continuous PC1 score for the 15-gene signature showed significant association with OS (hazard ratio [HR] = 1.23 and p = 0.0007). Inclusion of PC2 and PC3 did not improve the analysis appreciably (S3 Table). Since the ratio of negative versus positive lymph node or stage (IB-IIA) versus stage IIB was about 1:1, median of the PC1 score was used to dichotomize the PC1 score and therefore to classify patients into low and high risk groups (low and high PC1 score, respectively). Results showed the high PC1 group had a poorer survival than the low PC1 group (Fig 1: HR = 3.26 and p = 0.0002 by log-rank test; median survival time: 2.92 (35 months) and 1.25 (15 months) years for low and high PC1, respectively). Importantly, both continuous and dichotomized PC1 scores remained significantly associated with OS after adjustment for other covariates, including histology grade, AJCC stage, gender, and age at diagnosis (HR = 3.03 and p = 0.0015 for the median dichotomized PC1; HR = 1.19 and p = 0.0056 for the continuous PC1). In addition, the continuous PC1 score had a weak correlation with histology grade (p = 0.59), gender (p = 0.97), and AJCC TNM stage (p = 0.52) by one-way ANOVA or two-sample t-test (S3 Fig).

### Integration with the AJCC TNM staging system

**Fig 2:**
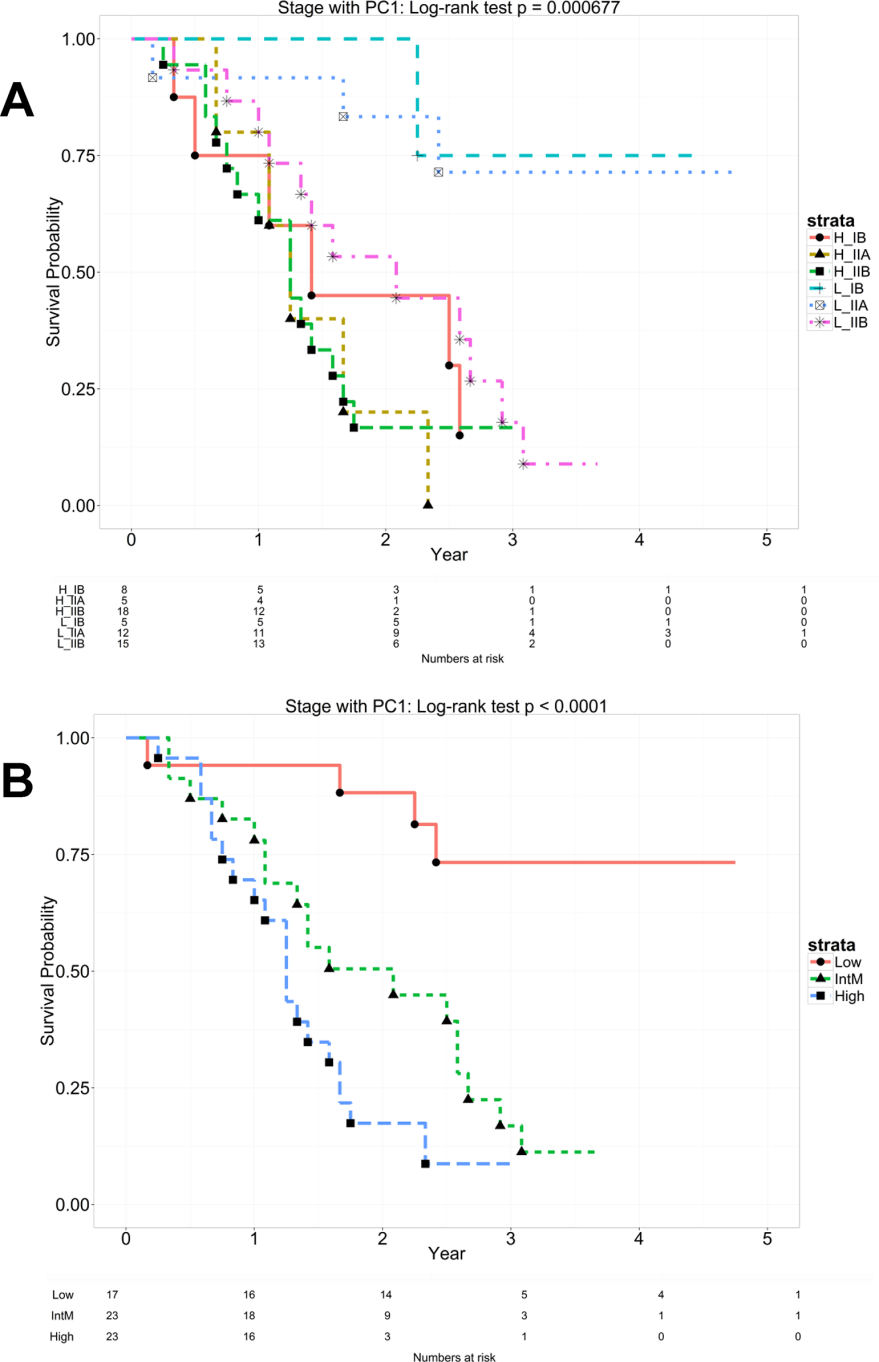
Integration of the 15-gene signature with the AJCC TNM staging system in the Moffitt cohort. A PC1 score was generated for each patient from the Moffitt cohort (n = 63) by principal component analysis to reflect the combined expression of the 15 genes. **(A)** The AJCC TNM staging variable and the median dichotomized PC1 score were used to classify patients into 6 groups: stage IB with low PC1: n = 5, stage IB with high PC1: n = 8, stage IIA with low PC1: n = 12, stage IIA with high PC1: n = 5, stage IIB with low PC1: n = 15, stage IIB with high PC1: n = 18. Kaplan–Meier curves of overall survival were shown in these six groups. **(B)** Regrouping of the 6 survival curves into 3 distinct clusters: (1) Low risk: Low PC1 with stage IB and IIA (n = 17; MST = never reach), (2) Intermediate risk: Low PC1 with stage IIB and high PC1 in stage IB (n = 23; MST = 2.08 years), (3) High-risk: High PC1 with stage IIA and IIB (n = 23; MST = 1.25 years). A statistically significant difference of the Kaplan–Meier survival curves between the groups was determined by the two-sided log-rank test. The number of patients at risk is listed below the survival curves. MST = median survival time.

To evaluate if the PC1 score could improve the AJCC TNM staging system for predicting better prognosis, the staging variable and the median dichotomized PC1 score were used to classify patients into 6 groups: (stage IB with low PC1: n = 5, stage IB with high PC1: n = 8, stage IIA with low PC1: n = 12, stage IIA with high PC1: n = 5, stage IIB with low PC1: n = 15, stage IIB with high PC1: n = 18). This classification yielded significant difference among the 6 Kaplan-Meier (KM) survival curves (p<0.001 by log-rank test; Fig 2A). Further analysis by stratification of the staging variable, the PC1 score was able to classify patients into low risk (low PC1) and high risk (high PC1) in each stage with a statistically significant difference at stage IIA (p<0.001; S4 Fig). Moreover, these 6 survival curves formed 3 distinct clusters (p<0.001; Fig 2B): (1) Low risk: Low PC1 with stage IB and IIA (median survival time: never reach), (2) Intermediate risk: Low PC1 with stage IIB and high PC1 in stage IB (median survival time: 2.08 years), (3) High-risk: High PC1 with stage IIA and IIB (median survival time: 1.25 years).

### External evaluation of the prognostic effect in the Stratford et al[12] localized PDAC study

There were 29 probesets matching to the 15-gene signature. Due to the difference of microarray platforms, gene level data were used for evaluation. The predetermined PC1 model from the Moffitt cohort was used to calculate the PC1 score for each patient in the Stratford et al cohort.

**Fig 3:**
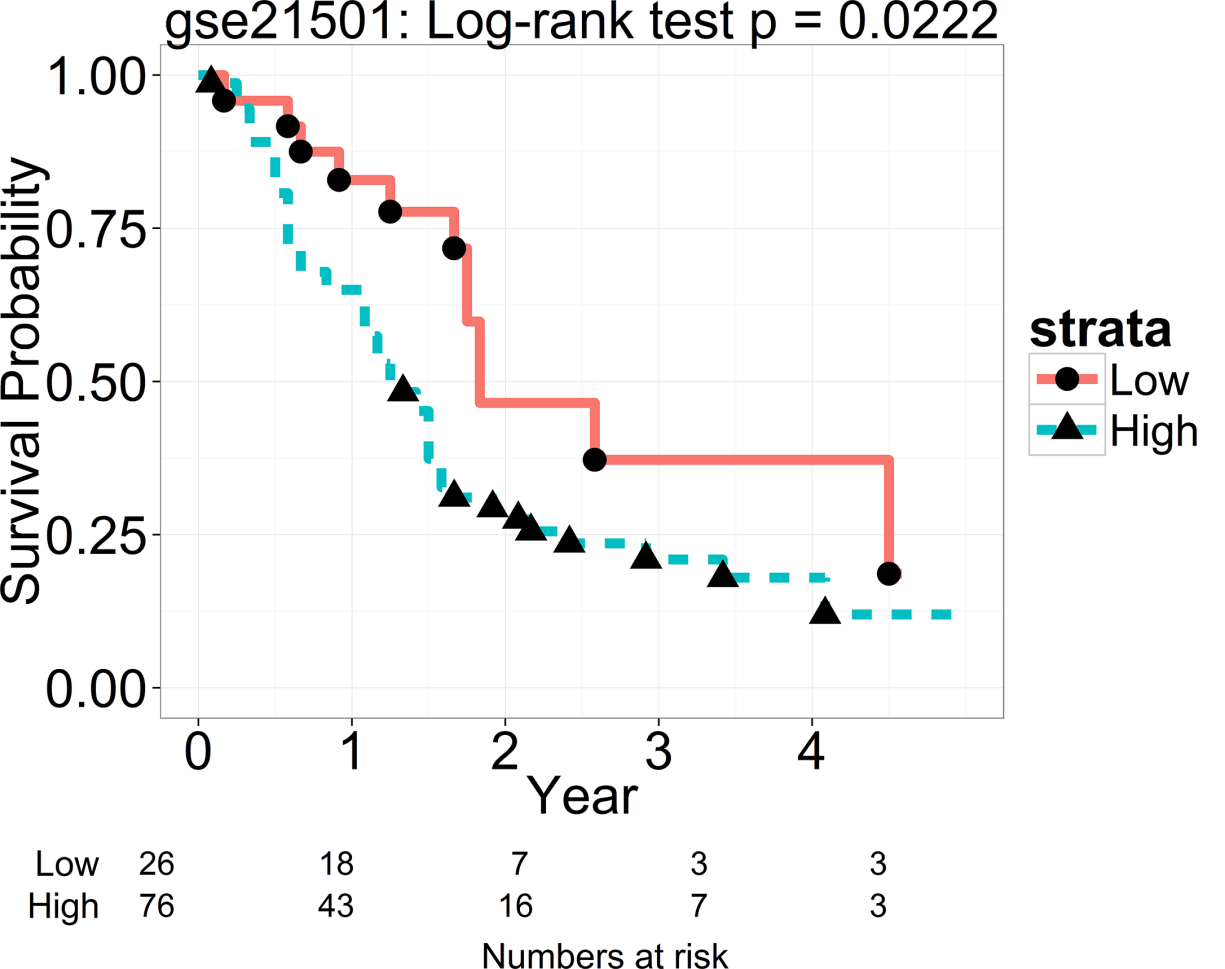
Prognostic value of the 15-gene signature in the Stratford et al cohort. A PC1 score was generated for each patient in the Stratford et al cohort (n = 102) using the loading coefficients of the first principal component from the Moffitt cohort. High and low PC1 groups were determined by the cutoff at the first quartile of the PC1 score to adjust for the distribution of the N staging. Kaplan–Meier curves of overall survival are shown in the two groups. A statistically significant difference of the Kaplan–Meier survival curves between the high and low PC1 groups was determined by the two-sided log-rank test. The number of patients at risk is listed below the survival curves.

Prior to determining the cutoff, we evaluated comparability of the two cohorts regarding patient characteristics (T and N staging variable for both cohorts). Results showed both cohorts had a comparable distribution of T stage (T1-2: 16 (25%) vs. T3: 47 (75%) in the Moffitt cohort; T1-2: 18 (18%) vs. T3-4: 80 (82%) in the Stratford et al cohort; p = 0.33 by Fisher exact test). However, distribution of the N staging variable was different between the two cohorts (N stage with negative lymph node: 28% in the Stratford et al cohort and 48% in the Moffitt cohort; p = 0.01). To adjust for the staging effect, the 1^st^ quartile (25^th^ percentile) of the PC1 score was used to classify patients into low and high PC1 in the Stratford et al cohort. A simulation study was conducted and showed that, even for an independent predictor, the cutoff guided by survival outcome-associated covariate (N staging) improves power compared to the cutoff derived from the training cohort (S4 Table). This classification yielded two significantly different survival curves with poor survival in the high PC1 group (HR = 2.07; p = 0.02; Fig 3) and maintained significance level (HR = 2.08; p = 0.025) after adjusting for the T and N staging variables. This PC1 classification had a comparable performance comparing to the N staging which also showed a significant association with OS (p = 0.029). There was a weak correlation of the PC1 score with T and N staging (p = 0.98 and 0.79, respectively). Further analysis using the N staging and the dichotomized PC1 to classify patients into 4 groups yielded significant differences among the 4 KM survival curves (p = 0.015 by log-rank test; S5A Fig) which formed 3 distinct clusters (p = 0.005; S5B Fig) similar to the ones in the Moffitt cohort: (1) Low risk: Low PC1 with N0 stage (median survival time: never reach), (2) Intermediate risk: Low PC1 with N1 stage and high PC1 in N0 stage (median survival time: 1.75 years), (3) High-risk: High PC1 with N1 stage (median survival time: 1.17 years).

### Likelihood as a non-random gene signature

Since a study indicated many gene signatures were a random noise signature[22], we conducted a resampling approach to evaluate the likelihood of the 15-gene signature as a random signature. Results showed a joint p value of 0.00001 to be a random noise signature in both datasets for the 15 genes (18 probesets). In each cohort, the probability of p values from the random signatures less than the observed p value (0.0007) in the Moffitt cohort was 0.00001. For the Stratford et al cohort, the probability was 0.01.

### NanoString validation

**Fig 4:**
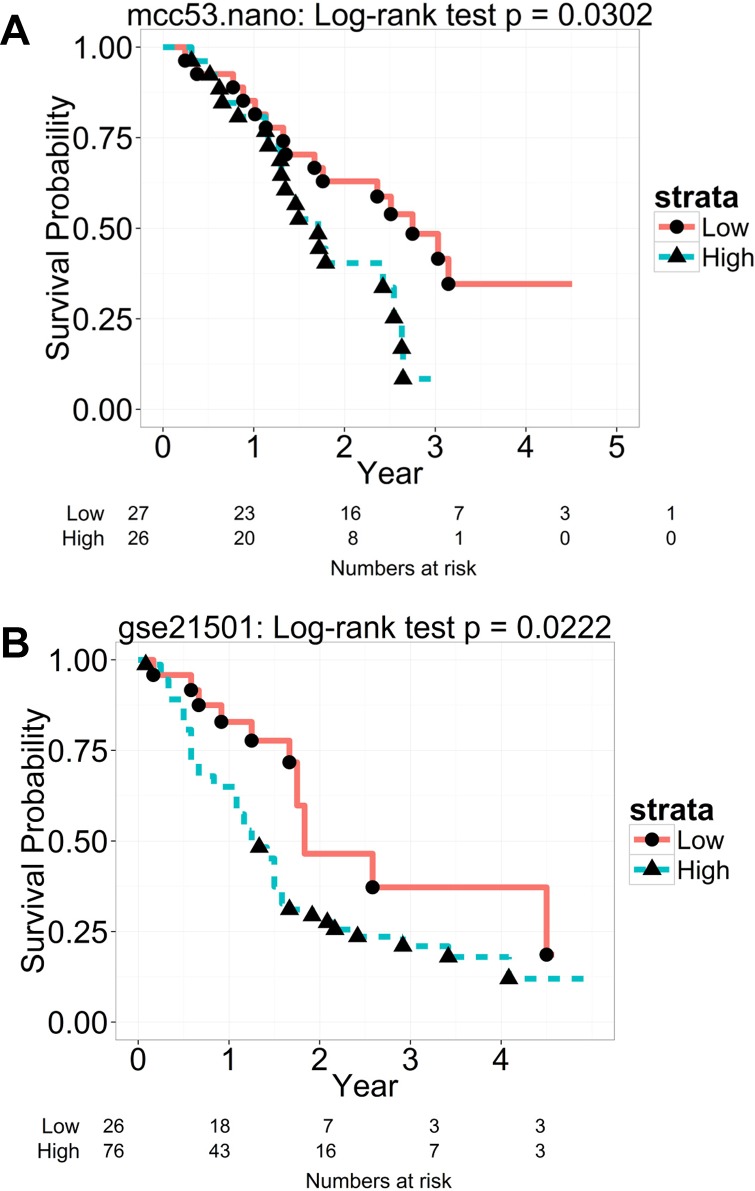
NanoString validation of the 15-gene signature. **(A)** By analyzing the NanoString gene expression data, a PC1 score was generated for each patient from the subset (n = 53) of the Moffitt cohort using principal component analysis to reflect the combined expression of the 15 genes. The median dichotomized PC1 score was used to classify patients into high and low PC1 groups. **(B)** For the Stratford et al cohort, the PC1 score was generated for each patient using the loading coefficients of the first principal component in the Moffitt cohort. High and low PC1 groups were determined by the cutoff at the first quartile of the PC1 score to adjust for the distribution of the N staging. Kaplan–Meier curves of overall survival are shown in the two groups. A statistically significant difference of the Kaplan–Meier survival curves between the high and low PC1 groups was determined by the two-sided log-rank test. The number of patients at risk is listed below the survival curves.

Gene expression by NanoString platform showed that the PC1 score of the 15-gene signature was associated with OS (p = 0.03; Fig 4A) in the Moffitt cohort. The predicted PC1 score also remained significantly associated with OS in the Stratford et al cohort (p = 0.02; Fig 4B). Pearson correlation analysis yielded 11 genes (73%) with a correlation (r)>0.6, 1 gene with r = 0.56, 2 genes r = 0.33–0.39, and 1 gene with r = 0.02 (S5 Table). Moreover, the PC1 loading coefficients were all positive with majority greater than 0.2 and comparable to the ones in the microarray data (S6 Table). The gene signature was further evaluated by sensitivity analysis based on different cutoff of correlation to select genes. Results showed that the PC1 based on the whole 15 genes or a subset of genes remained significant (p<0.05) in most cases (the non-significant one had a marginal p value of 0.069; S7 Table).

## Discussion

In the present study, we profiled resected primary tumors from early stage PDAC patients and developed a 15-gene signature based on overall survival. The signature was able to identify a low-risk subgroup of PDAC patients who were likely to survive longer than 2 years after surgery (median survival of 35 months) and a high-risk subgroup who likely survived less than 1.5 years (median survival of 15 months) (Fig 1). Importantly, the association of the signature with OS was independent of histology grade, AJCC TNM stage, gender, and age at diagnosis (p = 0.0015), indicating the potential clinical utility of the signature in predicting patients’ outcomes. Since the signature was a predictor of OS independent of TNM staging, we evaluated if incorporation of the signature into the TNM staging system could improve survival prediction. Our evaluation results showed the signature could be a complementary prognostic adjunct to the AJCC TNM staging system (Fig 2). Specifically, the signature was able to differentiate patients with poor survival and good survival in each stage, especially at stage IIA (S4B Fig). Furthermore, combination of the signature and staging formed three distinct risk groups from low, intermediate, to high risk. This pattern was observed in both cohorts (Fig 2B and S5B Fig), especially for the two subgroups: (a) patients having low signature score and negative lymph nodes classified in the low risk group and (b) patients with high signature score and positive lymph node grouped in the high risk group. This consistent result indicates that a new classification system, by integrating the signature into the TNM staging system, could be a useful prognostic tool for early stage PDAC patients.

From a personalized medicine point of view, the signature offers one potential strategy. That is, there is an unmet need to identify high-risk PDAC patients for alternative treatment management given the unsatisfactory outcome of the standard of care (surgery followed by adjuvant therapy)[23–28]. It is possible that these high-risk patients have developed micrometastases before surgery. In this case, neoadjuvant therapy may offer a better solution to treat micrometastases to prevent disease recurrence, or just to allow the patient and their disease to declare themselves and avoid unnecessary surgery. In PDAC patients with clinically positive lymph nodes, the signature identifies a subgroup with a high signature score. This high-risk group has a median survival time less than 15 months (Fig 2B and S5B Fig) which could be a potential candidate for the neoadjuvant therapy. However, future prospective validation cohort may shed light on the issue as the survival differences may be impacted by the fact that our analysis for intermediate risk patients includes N1 and N0 disease, and the survival differences in this group may be attributable to the presence of nodal disease.

Our literature review indicates that the 15-gene signature may have clinical relevance. Forty percent of the 15 genes (6 genes) showed their association with various clinical outcomes in pancreatic cancer by other studies[29–39]: (1) IGF2BP3: Elevation of mRNA and protein expression levels was significantly increased in PDAC tissues (but not in benign pancreatic tissues) and also in PDAC patients with poor survival[29, 30]; (2) KIF14: Over-expression of mRNA and protein levels was observed in all highly nerve-invasive pancreatic tumor cells, suggesting an association with perineural invasion[31]; (3) PPBP: The plasma level of this protein was higher in pancreatic cancer patients than in healthy controls and was independent of CA19-9 serum levels. The combination of this protein with CA19-9 improved the detection of pancreatic cancer at an early stage[39]; (4) SERPINB5: Increased mRNA expression was correlated with increased metastasis[36] and to progression of PDAC from pancreatic intraepithelial neoplasias (PanINs)[34], as well as associated with poor survival[34]; (5) SLC2A1: Positive expression by immunohistochemistry was associated with higher-grade PanIN and IPMN[37, 38], suggesting its involvement in the preinvasive phase of pancreatic carcinogenesis; (6) TMPRSS3: This gene was more highly expressed in pancreatic cancers than in non-neoplastic tissues[32], as well as associated with metastasis[33]. Moreover, the signature yielded a high degree of reproducibility from microarray to NanoString platform (S5 and S6 Tables) and the association with OS remained significant in the two cohorts (p<0.05; Fig 4). In addition, the resampling test showed the likelihood to be a false positive signature was rare (p = 0.00001). By taking these considerations together, we believe the 15-gene signature is not a random-noise signature, but a signature specific to PDAC as a prognostic tool to improve treatment decisions.

While various gene signatures have been developed in PDAC patients, the majority of them focus on separating between PDAC and normal pancreas or on the progression of dysplastic lesions[32, 40–42]. Only a few prognostic gene signatures[43–46] were developed. Most of them were with a small number of genes in the signatures due to the challenge of uniform global gene expression in PDAC. None of these signatures were overlapped with the 15-gene signature, except two genes listed in one gene signature[46]. Two signatures[12, 43] we evaluated did not overlap with the 15-gene signature and failed to show significant association with OS using the Moffitt cohort. This non-overlapped result is not uncommon. Different studies often yield different gene signatures within the same disease type. Many factors contribute to the inconsistent result, such as study design, characteristics of study cohort, and analysis approaches. Thus, utilization of publically available microarray data as external validation cohorts is not straightforward. We understand that the gene signature developed in our cohort is unlikely to be the best in other cohorts because it was not trained and optimized in external cohorts. However, if it shows an overall significant level in the external cohorts, the chance as a false positive gene signature becomes small. For the 15-gene signature, while it was not in the list of the optimal gene set in the Stratford et al cohort (data not shown), the p value showed an overall significant level (p = 0.02). In addition, it passes the noise signature test. Therefore, the 15-gene signature is unlikely a false positive signature.

Our study has some limitations. We have shown the prognostic value of the 15-gene signature using the Moffitt cohort and one publically available microarray dataset in PDAC. However, in order to be considered as a personalized medicine strategy for clinical decision making, validation of the 15-gene signature in a larger independent dataset is needed. Successful validation will advance the 15-gene signature to the next level for the analytical and clinical validity. We plan to evaluate the 15-gene signature and complete a large-scale validation using formalin-fixed paraffin embedded (FFPE) tissues from Total Cancer Care [47, 48] collected at the Moffitt Cancer Center. Since FFPE tissues are routinely collected and stored long-term on pancreatic cancer patients and Nanostring platform performs quite well with FFPE tissue[49], if the signature using the Nanostring platform could be validated in FFPE tissues, the signature would be a great clinical utility for broad application in personalizing treatment care.

Another issue is determination of the cutoff to define low and high risk groups in the validation cohort. Ideally, to avoid over-fitting, the cutoff for the validation cohort should be derived from the predetermined model in the training cohort, especially for an independent predictor. However, each cohort may have a different distribution of patient characteristics and the microarray experiments may not be implemented by the same procedure with the same platform. Therefore, it has been challenging to implement such an ideal formula. Various modifications were used to address the issue, such as the use of median cutoff in each cohort (training and validation cohorts)[50]. In this study, we used a survival-outcome-associated covariate to guide the cutoff of the signature score in both training and validation cohorts, rather than an arbitrary cutoff, such as median or quartile. Simulation results indicate, even for an independent predictor, the cutoff guided by outcome-associated covariate improves power compared to the cutoff derived from the training cohort (S4 Table)

In summary, this 15-gene signature could be useful to improve prediction of OS in PDAC patients. This is a potential prognostic tool to allow risk-adapted stratification of pancreatic cancer patients into personalized treatment protocols, thereby improving the currently poor clinical outcomes of these patients. Future prospective studies are needed to determine if the 15-gene signature can be used clinically to benefit early stage PDAC patients.

## Supporting Information

S1 Fig Flow chart of data analysis. (PDF)

S2 Fig Principal component analysis of the 15-gene signature. (PDF)

S3 Fig Association of the 15-gene signature with histology grade, gender, and TNM staging system in the Moffitt cohort. (PDF)

S4 Fig Analysis of the association between the 15-gene signature and overall survival by TNM stage in the Moffitt cohort. (PDF)

S5 Fig Integration of the 15-gene signature with the AJCC TNM staging system in the Stratford et al cohort. (PDF)

S1 Table Housekeeping genes (19 genes) for NanoString validation. (PDF)

S2 Table Univariate analysis of the 15-gene signature in microarray data at the Moffitt cohort. (PDF)

S3 Table Analysis of the first three principal components of the 15-gene signature (18 probesets) and 689 probesets after sparse PCA filtering for the association with OS using Cox proportional hazards model in the Moffitt cohort (n = 63). (PDF)

S4 Table Simulation study to evaluate the cutoff based on survival-outcome-associated covariate. (PDF)

S5 Table Correlation analysis of microarray and NanoString data at the Moffitt cohort (N = 53). (PDF)

S6 Table PC1 loading coefficients of the 15-gene signature. (PDF)

S7 Table NanoString validation by various cutoffs of Pearson correlation for gene selection. (PDF)
