# Supplementary figures and images for: Prognostic Fifteen-Gene Signature for Early Stage Pancreatic Ductal Adenocarcinoma
Source: PLoS One. 2015 Aug 6;10(8):e0133562. doi: 10.1371/journal.pone.0133562 (PMC4527782; doi:10.1371/journal.pone.0133562)

**S1 Fig.** Flow chart of data analysis

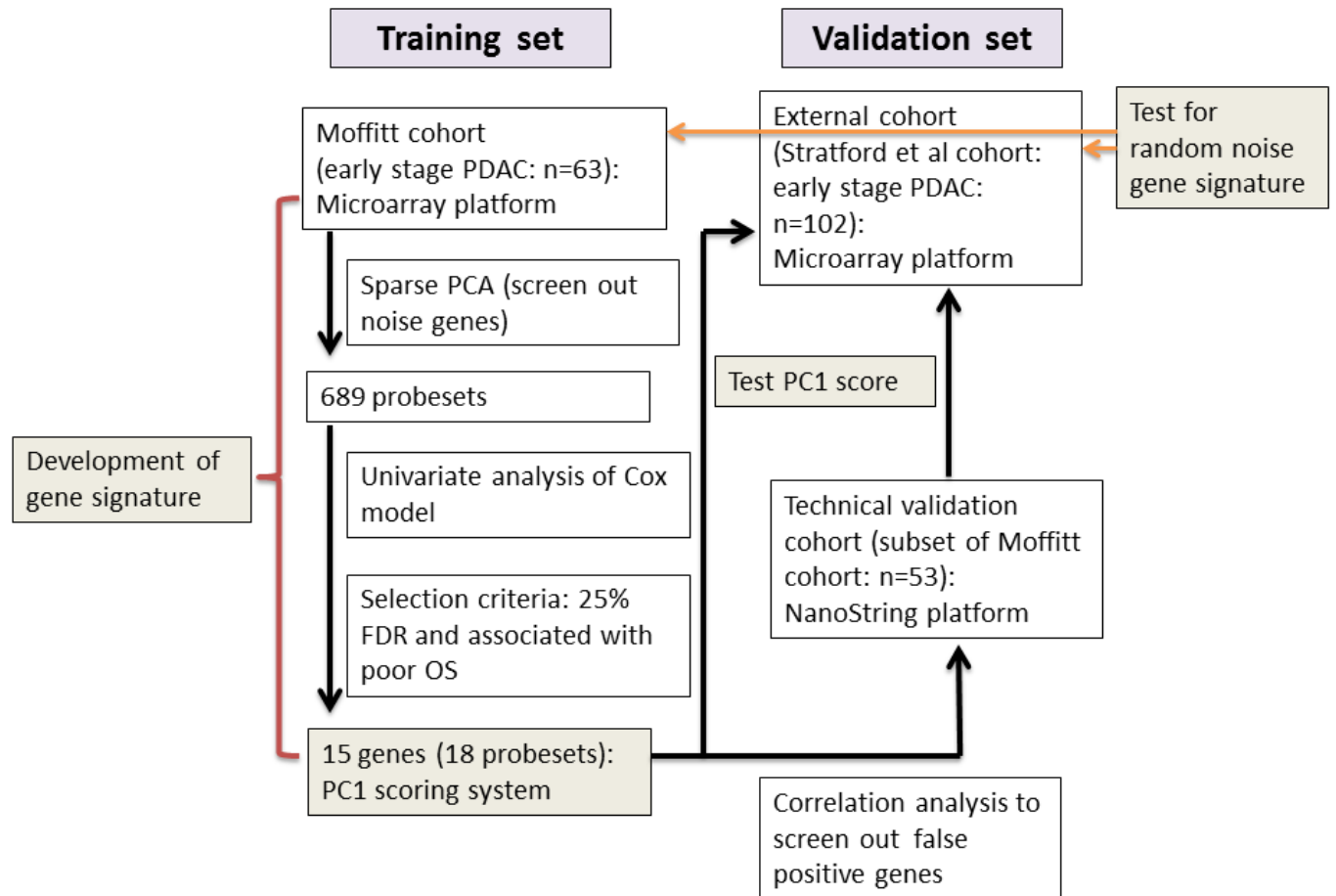

Supplement: S1 Fig — (PDF) [file pone.0133562.s001.pdf]
