# Supplementary material for: Prognostic Fifteen-Gene Signature for Early Stage Pancreatic Ductal Adenocarcinoma
Source: PLoS One. 2015 Aug 6;10(8):e0133562. doi: 10.1371/journal.pone.0133562 (PMC4527782; doi:10.1371/journal.pone.0133562)

S2 Fig. Principal component analysis of the 15-gene signature. Variation of the first five principal components in the two datasets: **A**) Moffitt cohort and **B**) Stratford et al cohort.

(A)

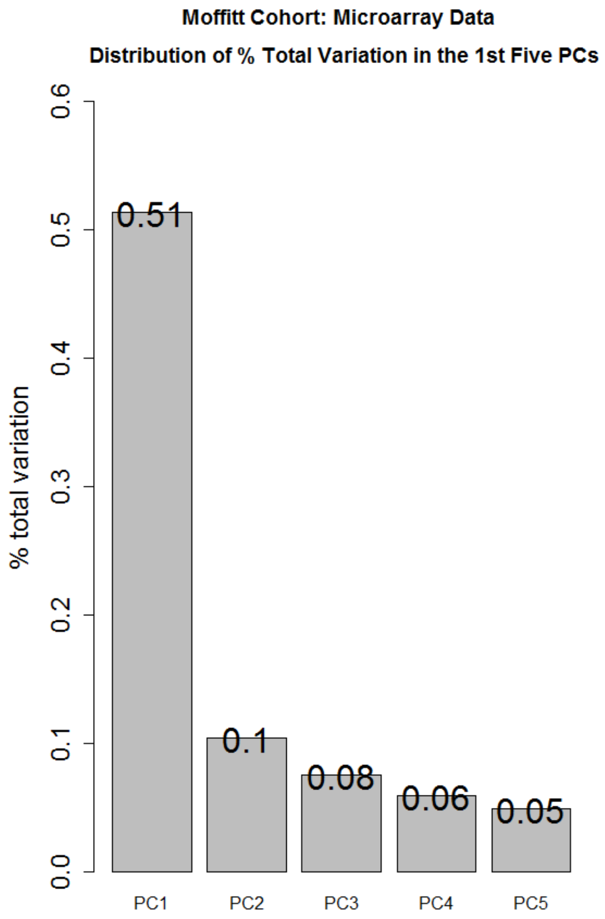

(B)

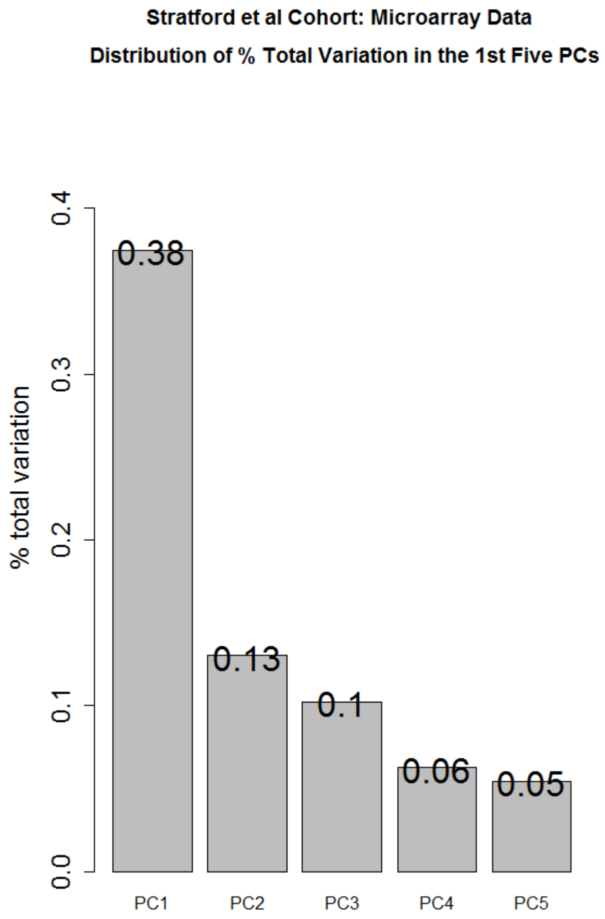

Supplement: S2 Fig — (PDF) [file pone.0133562.s002.pdf]
