# Supplementary material for: Prognostic Fifteen-Gene Signature for Early Stage Pancreatic Ductal Adenocarcinoma
Source: PLoS One. 2015 Aug 6;10(8):e0133562. doi: 10.1371/journal.pone.0133562 (PMC4527782; doi:10.1371/journal.pone.0133562)

S3 Fig. Association of the 15-gene signature with histology grade, gender, and TNM staging system in the Moffitt cohort. The PC1 score was calculated for patients from the Moffitt cohort for whom data on **A**) histology grade, **B**) gender, or **C**) TNM stage were available (n = 63). Boxplot was used to display distribution of the malignancy-risk score within each group. The bottom and top of each box are the lower and upper quartiles, respectively. The black band near the middle of the box is the median. The extreme of the lower whisker represents the lower quartile minus 1.5 times the interquartile range and the extreme of the higher whisker is the upper quartile plus 1.5 times the interquartile range. Any data points beyond the extremes of the whiskers are indicated by empty circles as outliers. One-way ANOVA or two-sample t-test was used to determine any association between the continuous PC1 score and histology grade, gender, and TNM stage. All statistical tests were two-sided.

(A)

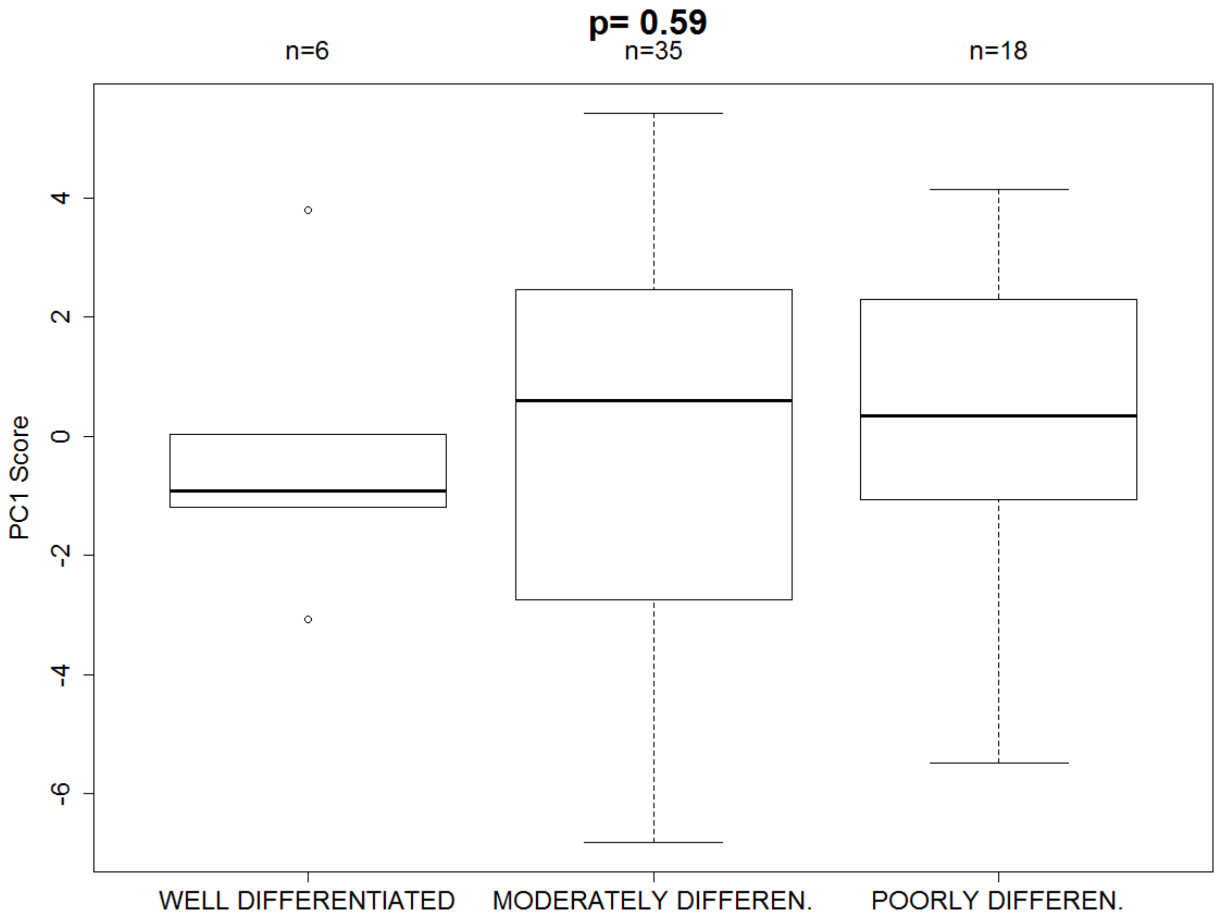

(B)

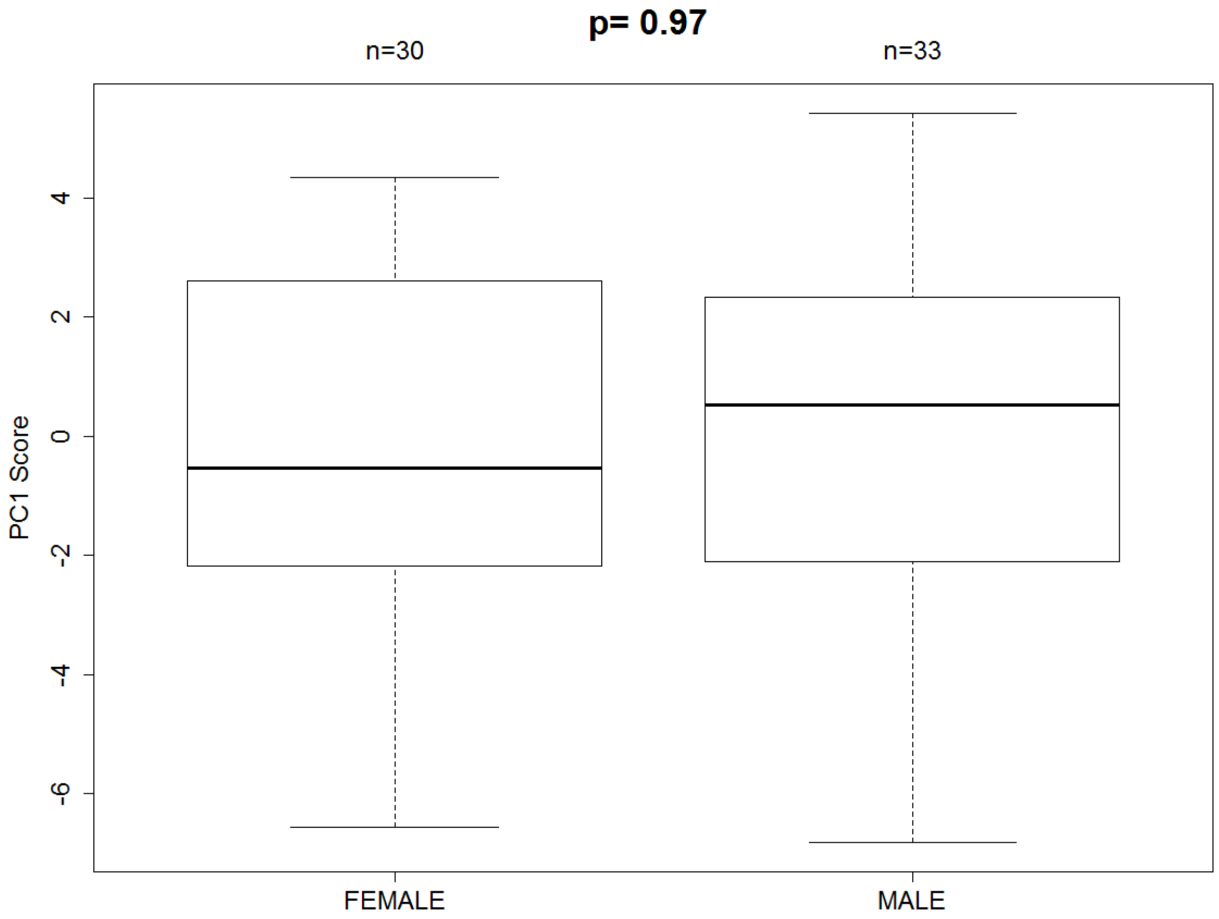

(C)

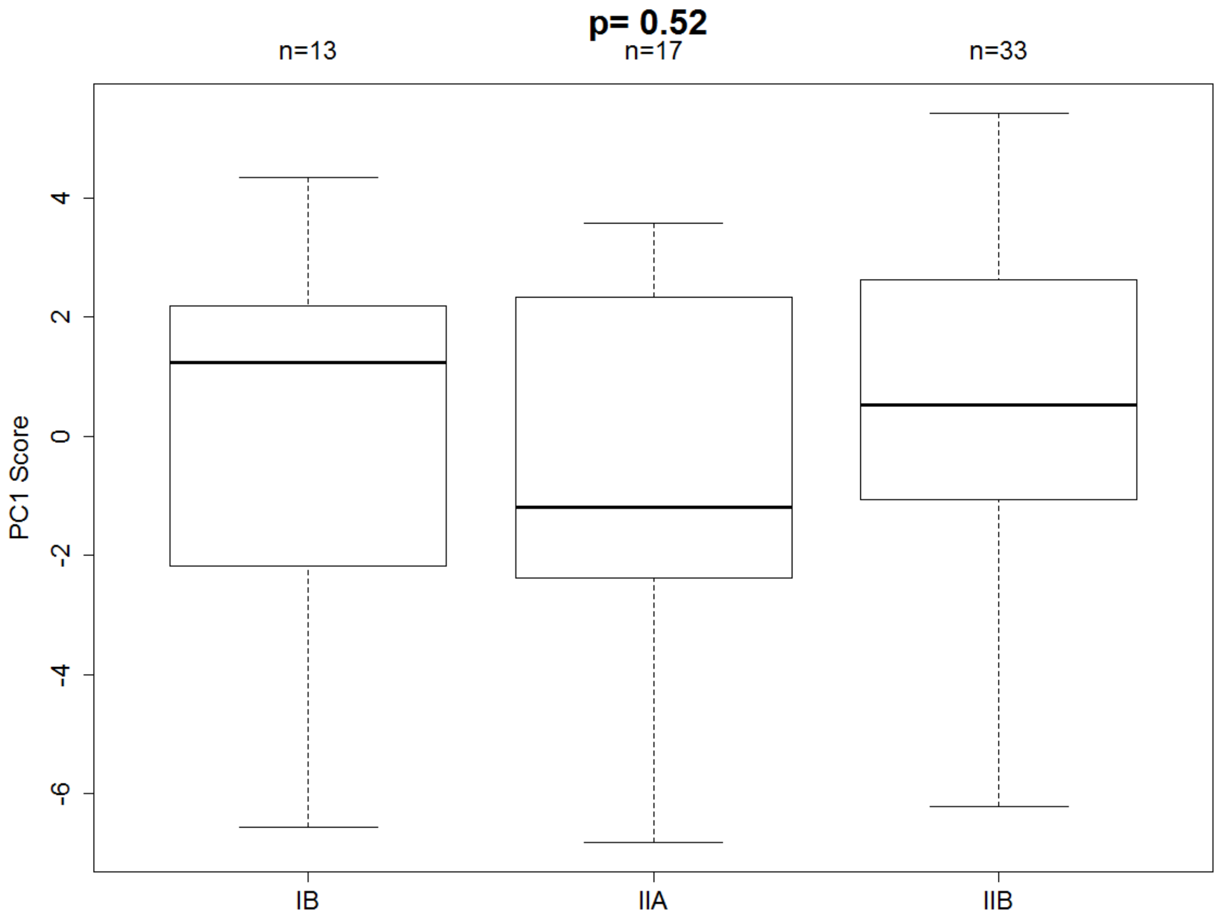

Supplement: S3 Fig — (PDF) [file pone.0133562.s003.pdf]
