# Supplementary material for: Prognostic Fifteen-Gene Signature for Early Stage Pancreatic Ductal Adenocarcinoma
Source: PLoS One. 2015 Aug 6;10(8):e0133562. doi: 10.1371/journal.pone.0133562 (PMC4527782; doi:10.1371/journal.pone.0133562)
Supplement: S4 Fig — (PDF) [file pone.0133562.s004.pdf]

S4 Fig. Analysis of the association between the 15-gene signature and overall survival by TNM stage in the Moffitt cohort. Kaplan–Meier curves of overall survival for patients from the Moffitt cohort stratified by TNM stage: **A)** IB, **B)** IIA, and **C)** IIB. A PC1 score was generated for each patient by principal component analysis to reflect the combined effect of the 15 genes. High and low PC1 groups were determined on the basis of a median split. A statistically significant difference in the Kaplan–Meier survival curves between the low and high PC1 groups for patients within each TNM stage was determined by the two-sided log-rank test.

(A)

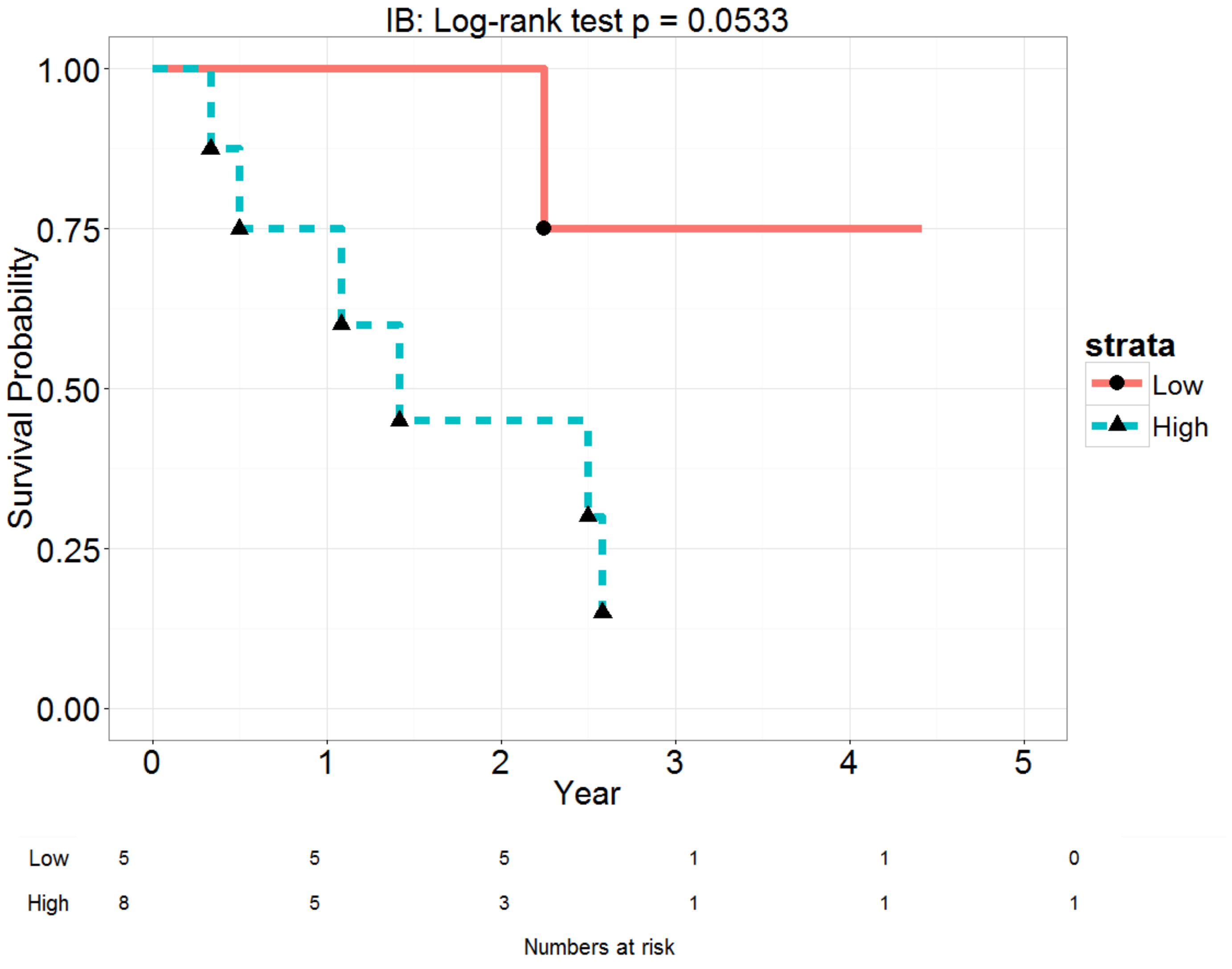

(B)

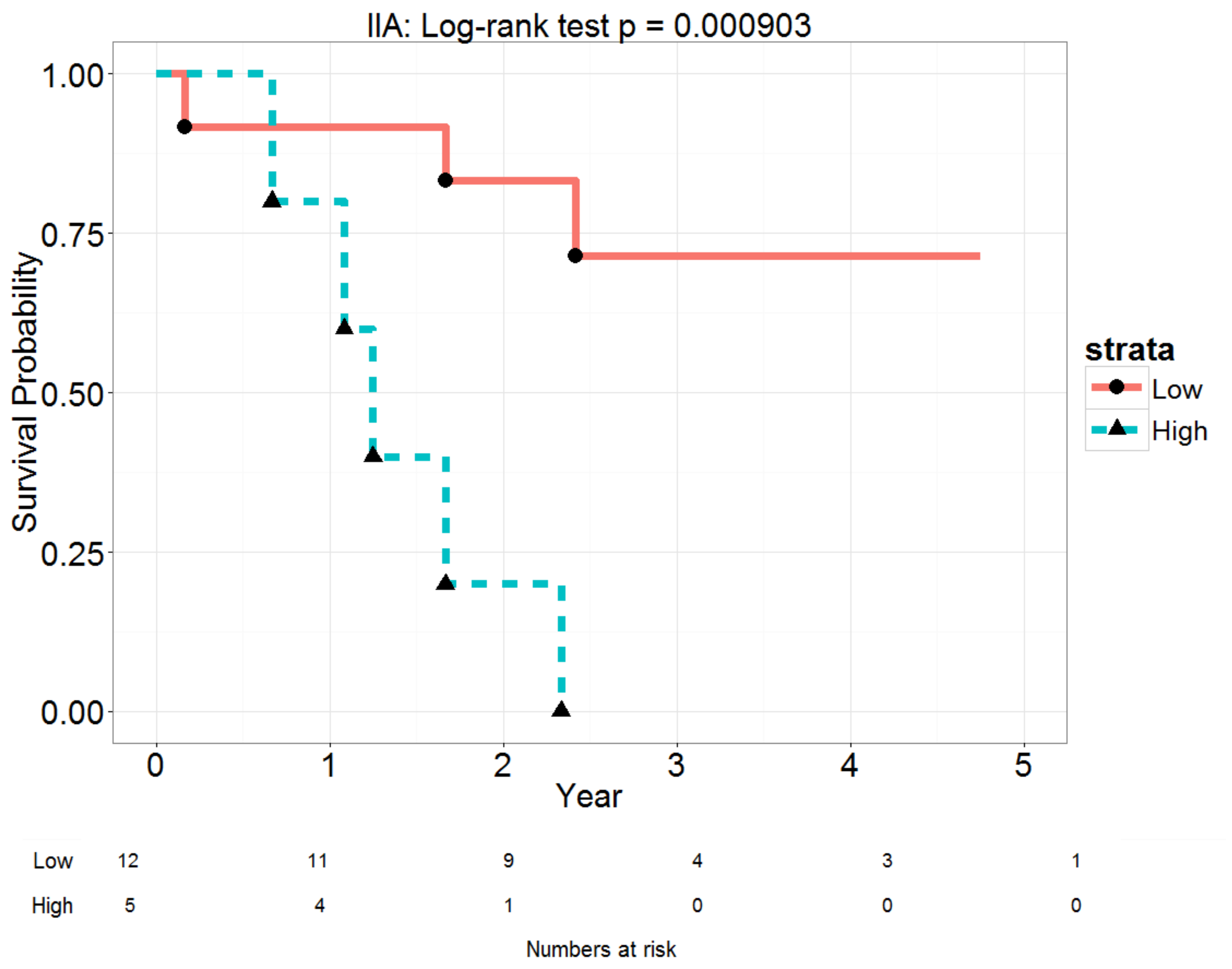

IIA: Log-rank test p = 0.000903
Survival Probability
1.00
0.75
0.50
0.25
0.00
0
1
2
3
4
5
Year
strata
Low
High
Low 12 11 9 4 3 1
High 5 4 1 0 0 0
Numbers at risk

(C)

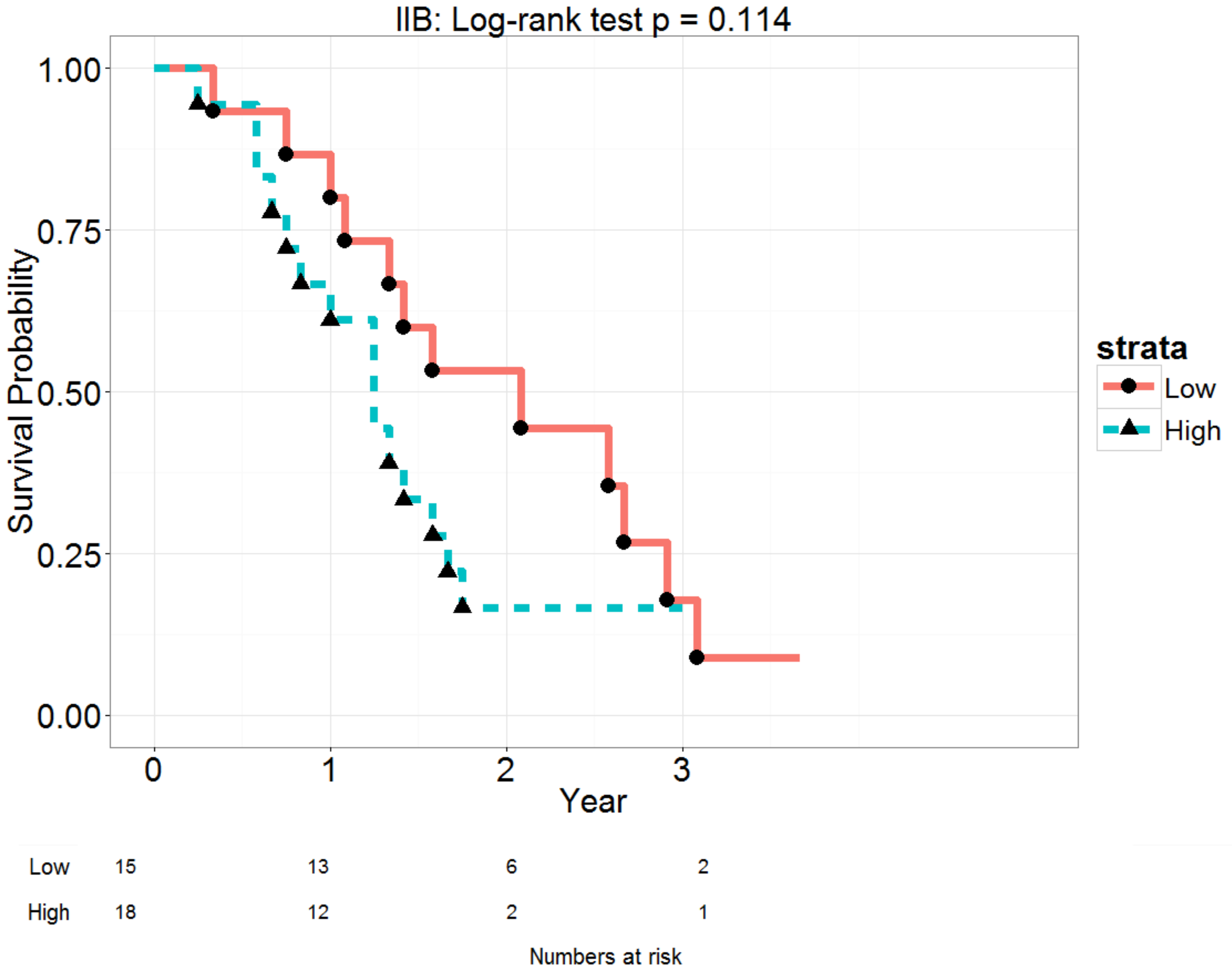

IIB: Log-rank test p = 0.114
Survival Probability
1.00
0.75
0.50
0.25
0.00
0
1
2
3
Year
strata
Low
High
Low 15 13 6 2
High 18 12 2 1
Numbers at risk
