# Supplementary material for: Prognostic Fifteen-Gene Signature for Early Stage Pancreatic Ductal Adenocarcinoma
Source: PLoS One. 2015 Aug 6;10(8):e0133562. doi: 10.1371/journal.pone.0133562 (PMC4527782; doi:10.1371/journal.pone.0133562)
Supplement: S5 Fig — (PDF) [file pone.0133562.s005.pdf]

S5 Fig. Integration of the 15-gene signature with the AJCC TNM staging system in the Stratford et al cohort. **(A)** The N staging variable and the dichotomized PC1 score were used to classify patients into 4 groups: N0 with low PC1: n=6, N0 with high PC1: n=22, N1 with low PC1: n=19, N1 with high PC1: n=54. Kaplan–Meier curves of overall survival were shown in these four groups. **(B)** Regrouping of the 4 survival curves into 3 distinct clusters: (1) Low risk: Low PC1 with N0 stage (MST: never reach), (2) Intermediate risk: Low PC1 with N1 stage and high PC1 in N0 stage (MST: 1.75 years), (3) High-risk: High PC1 with N1 stage (MST: 1.17 years). A statistically significant difference of the Kaplan–Meier survival curves between the groups was determined by the two-sided log-rank test. The number of patients at risk is listed below the survival curves. MST = median survival time.

(A)

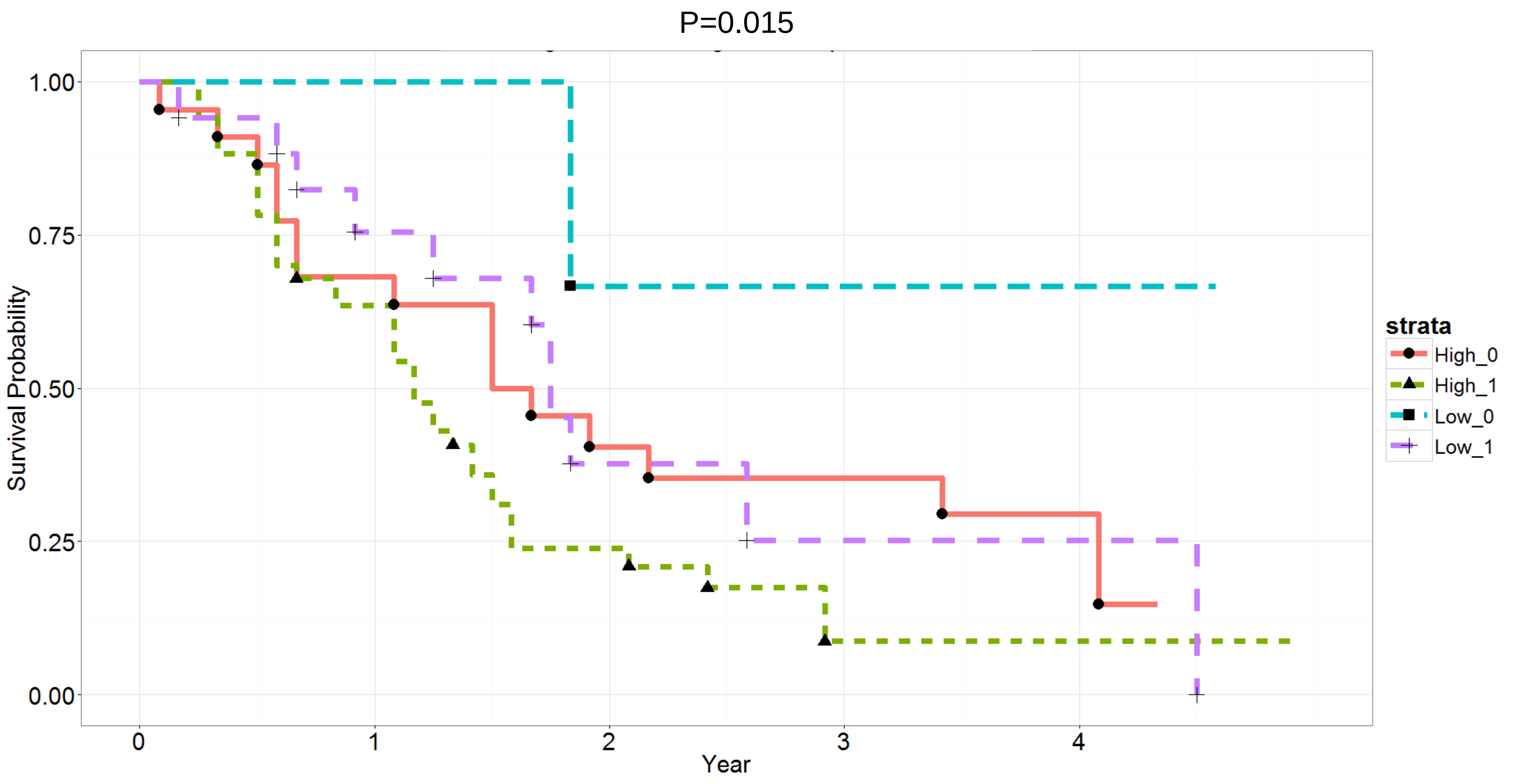

High_0	22	15	8	6	2
High_1	54	28	8	1	1
Low_0	6	6	2	2	2
Low_1	19	11	5	1	1

Numbers at risk

(B)

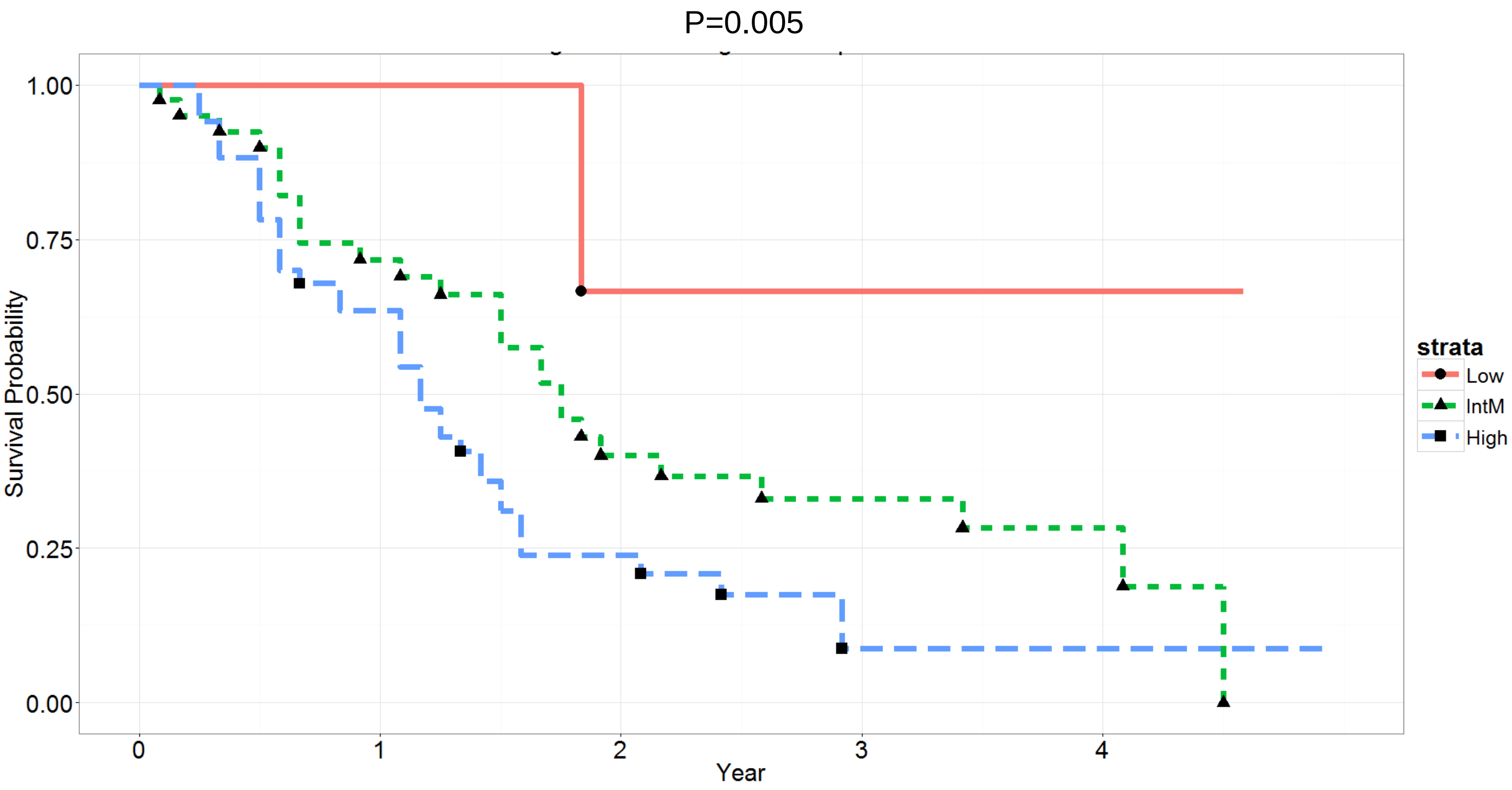

Low	6	6	2	2	2
IntM	41	26	13	7	3
High	54	28	8	1	1

Numbers at risk
