# Supplementary material for: Prognostic Fifteen-Gene Signature for Early Stage Pancreatic Ductal Adenocarcinoma
Source: PLoS One. 2015 Aug 6;10(8):e0133562. doi: 10.1371/journal.pone.0133562 (PMC4527782; doi:10.1371/journal.pone.0133562)
Supplement: S1 Table — (PDF) [file pone.0133562.s006.pdf]

S1 Table. Housekeeping genes (19 genes) for NanoString validation

Name	Accession
TOMM20	NM_014765.2
UBE3A	NM_000462.2
ATXN1L	NM_001137675.2
JUND	NM_005354.4
KY	NM_178554.4
CCDC73	NM_001008391.3
PFDN1	NM_002622.4
RPS3	NM_001005.4
WNK2	NM_006648.3
SDHAF2	NM_017841.2
MAPK14	NM_001315.1
ZCCHC14	NM_015144.2
RPS25	NM_001028.2
NOXA1	NM_006647.1
TPT1	NM_003295.2
RAB3GAP1	NM_001172435.1
RPS19	NM_001022.3
RPL17	NM_000985.3
RPL36	NM_033643.2
