# Supplementary material for: Prognostic Fifteen-Gene Signature for Early Stage Pancreatic Ductal Adenocarcinoma
Source: PLoS One. 2015 Aug 6;10(8):e0133562. doi: 10.1371/journal.pone.0133562 (PMC4527782; doi:10.1371/journal.pone.0133562)
Supplement: S2 Table — (PDF) [file pone.0133562.s007.pdf]

S2 Table. Univariate analysis of the 15-gene signature in microarray data at the Moffitt cohort.

Probeset id	Gene symbol	HR	log-rank p	log-rank adjusted p value
merck-NM_014070_a_at	C6orf15	2.2258	0.0115	0.2138
merck-AI921300_a_at	CAPN8	2.4823	0.0034	0.1122
merck-ENST00000377383_at	HIST1H3H	2.4266	0.0046	0.1214
merck-NM_006547_at	IGF2BP3	2.3804	0.0048	0.1233
merck-BX648488_s_at	IGF2BP3	2.7127	0.0013	0.0818
merck2-BC098582_at	KIF14	2.2817	0.0085	0.1892
merck2-NM_005554_at	KRT6A	3.9158	0	0.0126
merck-ENST00000269518_a_at	PMAIP1	2.4264	0.0041	0.1188
merck-NM_002704_at	PPBP	2.2515	0.0094	0.2021
merck-BI768238_a_at	RTKN2	3.1157	0.0003	0.0541
merck-NM_003843_a_at	SCEL	2.5678	0.0027	0.1122
merck-NM_002639_at	SERPINB5	2.567	0.0029	0.1122
merck-BQ217236_a_at	SERPINB5	2.4586	0.0043	0.1188
merck-AF086216_at	SERPINB5	2.1981	0.0113	0.2138
merck-NM_006516_at	SLC2A1	2.8228	0.001	0.076
merck-BX640973_at	SLC45A3	2.7577	0.0011	0.076
merck2-NM_032405_at	TMPRSS3	2.5135	0.0029	0.1122
merck-DQ343132_s_at	UCA1	2.7587	0.0011	0.076
