# Supplementary material for: Prognostic Fifteen-Gene Signature for Early Stage Pancreatic Ductal Adenocarcinoma
Source: PLoS One. 2015 Aug 6;10(8):e0133562. doi: 10.1371/journal.pone.0133562 (PMC4527782; doi:10.1371/journal.pone.0133562)
Supplement: S3 Table — (PDF) [file pone.0133562.s008.pdf]

S3 Table. Analysis of the first three principal components of the 15-gene signature (18 probesets) and 689 probesets after sparse PCA filtering for the association with OS using Cox proportional hazards model in the Moffitt cohort (n=63)

	Model	Log-rank test statistics	Degree of freedom	P value
15-gene signature (18 probesets)	PC1	12.02	1	0.0005
	PC1+PC2	13.74	2	0.001
	PC1+PC2+PC3	16.40	3	0.0009
The PC1 accounted for 51% total variation while PC2 and PC3 explained only 10% and 8% variation, respectively				
689 probesets	PC1	2.52	1	0.11
	PC1+PC2	2.73	2	0.26
	PC1+PC2+PC3	2.74	3	0.43
The PC1 accounted for 45% total variation while PC2 and PC3 explained only 9% and 6% variation, respectively				
