# Supplementary material for: Prognostic Fifteen-Gene Signature for Early Stage Pancreatic Ductal Adenocarcinoma
Source: PLoS One. 2015 Aug 6;10(8):e0133562. doi: 10.1371/journal.pone.0133562 (PMC4527782; doi:10.1371/journal.pone.0133562)
Supplement: S4 Table — (PDF) [file pone.0133562.s009.pdf]

S4 Table. Simulation study to evaluate the cutoff based on survival-outcome-associated covariate.

Simulation design: Assume two populations: low and high risk groups with median survival time (MST) of 30 months and 10 months (HR=3), respectively. Survival time is generated for both groups based on exponential distribution. For simplicity, random censoring (30% censoring rate) is used to generate censor status. Two independent covariates (X1 and X2) are simulated based on normal distribution. For low risk group, X1~N(0,1) and X2~N(0,1). X1 and X2 are independent. For high risk group, X1~N(3,1) and X2~N(2,1). X1 and X2 are independent. This setting will create association of X1 and X2 with risk status (low and high), as well as with survival data. Both data are combined (low and high risk groups) and the X2 variable is dichotomized at the cutoff of percentile of ($n_{(low\text{-}risk)}$/($n_{(low\text{-}risk)}$+ $n_{(high\text{-}risk)}$) where $n_{(low\text{-}risk)}$ and $n_{(high\text{-}risk)}$ represent the sample size for low and high risk groups, respectively. This setting is to mimic the N stage (N0 and N1) to have association with survival outcome and independent of X1 (mimic PC1). With this design, we simulate two cohort datasets: training cohort and validation cohort with an equal total sample size of 100 patients. For the training cohort, the ratio of sample size is 1:1 for low and high risk groups (n=50 per group). For the validation cohort, the ratio is 1:3 (n=25 and 75 for low and high risk groups, respectively).

At each simulation, for the training cohort, we first dichotomize the X2 variable into N0 and N1 two subgroups. Then we scale the X1 variable and dichotomize it into low and high score groups based on the cutoff of percentile of ($n_{(N0)}$/($n_{(N0)}$+ $n_{(N1)}$). Two Cox models are performed: (a) X1 as the only covariate and (b) X1 and the binary X2 as two covariates (evaluation of independent predictor, X1, in multivariate analysis). P value for X1 is collected for both models.

For the validation cohort, we first dichotomize the X2 variable into N0 and N1 two subgroups. Then we scale the X1 variable. Two approaches for dichotomization of X1 are used: (1) based on the binary X2 variable: X1 is dichotomized into low and high score groups based on the cutoff of percentile of ($n_{(N0)}$/($n_{(N0)}$+ $n_{(N1)}$). (2) based on the training cohort's cutoff: X1 is dichotomized into low and high score groups based on the cutoff derived from the training cohort. Two Cox models are performed for each dichotomization approach: (a) X1 as the only covariate and (b) X1 and the binary X2 as two covariates. P value for X1 is collected for both models in each dichotomization approach.

The significance level (power) is defined as proportion of all the 4 p values of X1 <0.05 (two from the training cohort and two from the validation cohort). We compare the power between the two dichotomization (cutoff) approaches. Over 10,000 simulations, results indicate the cutoff guided by survival-outcome-associated covariate gives a better power than the cutoff derived from the training cohort. Our comment is that, even for an independent predictor, determination of the cutoff for validation cohort needs to be exercised carefully due to many unknown confounding factors such that validation cohort is not always comparable to the training cohort.

Power	HR						
	2	3	4	5	6	7	8
Cutoff guided by survival-outcome-associated covariate	0.08	0.35	0.55	0.68	0.77	0.82	0.85
Cutoff derived from the training cohort	0.07	0.30	0.48	0.61	0.70	0.76	0.80
