# Supplementary material for: Prognostic Fifteen-Gene Signature for Early Stage Pancreatic Ductal Adenocarcinoma
Source: PLoS One. 2015 Aug 6;10(8):e0133562. doi: 10.1371/journal.pone.0133562 (PMC4527782; doi:10.1371/journal.pone.0133562)
Supplement: S5 Table — (PDF) [file pone.0133562.s010.pdf]

S5 Table. Correlation analysis of microarray and NanoString data at the Moffitt cohort (N=53).

Gene Name	Correlation between Micorarray and NanoString
IGF2BP3	0.90
SERPINB5	0.88
UCA1	0.87
SCEL	0.87
PPBP	0.83
SLC2A1	0.81
KIF14	0.77
RTKN2	0.72
CAPN8	0.66
C6orf15	0.66
TMPRSS3	0.61
KRT6A	0.56
SLC45A3	0.39
HIST1H3H	0.33
PMAIP1	0.02
