# Supplementary material for: Prognostic Fifteen-Gene Signature for Early Stage Pancreatic Ductal Adenocarcinoma
Source: PLoS One. 2015 Aug 6;10(8):e0133562. doi: 10.1371/journal.pone.0133562 (PMC4527782; doi:10.1371/journal.pone.0133562)
Supplement: S6 Table — (PDF) [file pone.0133562.s011.pdf]

S6 Table. PC1 loading coefficients of the 15-gene signature.

			Gene Level: PC1 loading coefficients	
Probeset ID	PC1 loading coefficients	Gene Name	Microarray	NanoString
merck-NM_014070_a_at	0.157	C6ORF15	0.183	0.163
merck-AI921300_a_at	0.178	CAPN8	0.194	0.243
merck-ENST00000377383_at	0.220	HIST1H3H	0.252	0.183
merck-NM_006547_at	0.234	IGF2BP3	0.260	0.316
merck-BX648488_s_at	0.237			
merck2-BC098582_at	0.261	KIF14	0.289	0.321
merck2-NM_005554_at	0.247	KRT6A	0.271	0.183
merck-ENST00000269518_a_at	0.247	PMAIP1	0.277	0.052
merck-NM_002704_at	0.157	PPBP	0.183	0.139
merck-BI768238_a_at	0.256	RTKN2	0.290	0.338
merck-NM_003843_a_at	0.243	SCEL	0.275	0.287
merck-NM_002639_at	0.271	SERPINB5	0.296	0.312
merck-BQ217236_a_at	0.274			
merck-AF086216_at	0.269			
merck-NM_006516_at	0.281	SLC2A1	0.311	0.333
merck-BX640973_at	0.271	SLC45A3	0.304	0.296
merck2-NM_032405_at	0.208	TMPRSS3	0.233	0.297
merck-DQ343132_s_at	0.170	UCA1	0.200	0.202
