# Supplementary material for: Prognostic Fifteen-Gene Signature for Early Stage Pancreatic Ductal Adenocarcinoma
Source: PLoS One. 2015 Aug 6;10(8):e0133562. doi: 10.1371/journal.pone.0133562 (PMC4527782; doi:10.1371/journal.pone.0133562)
Supplement: S7 Table — (PDF) [file pone.0133562.s012.pdf]

S7 Table. NanoString validation by various cutoffs of Pearson correlation for gene selection.

NanoString Validation		p value	
Correlation	Number of genes selected by correlation	Moffitt Cohort (n=53): NanoString	Stratford cohort (n=102): Microarray
>0	15	0.03	0.02
>0.1	14	0.006	0.02
>0.5	12	0.007	0.044
>0.6	11	0.01	0.044
>0.7	8	0.03	0.069
>0.8	6	0.006	0.039
